# A computational role for bistability and traveling waves in motor cortex

**DOI:** 10.3389/fncom.2012.00067

**Published:** 2012-09-11

**Authors:** Stewart Heitmann, Pulin Gong, Michael Breakspear

**Affiliations:** ^1^School of Psychiatry, The University of New South WalesSydney, NSW, Australia; ^2^The Black Dog InstituteSydney, NSW, Australia; ^3^School of Physics, The University of SydneyNSW, Australia; ^4^Faculty of Medicine, The University of SydneyNSW, Australia; ^5^Queensland Institute of Medical ResearchBrisbane, QLD, Australia; ^6^Royal Brisbane and Women's HospitalBrisbane, QLD, Australia

**Keywords:** motor cortex, synchrony, traveling waves, beta oscillations, bistability

## Abstract

Adaptive changes in behavior require rapid changes in brain states yet the brain must also remain stable. We investigated two neural mechanisms for evoking rapid transitions between spatiotemporal synchronization patterns of beta oscillations (13–30 Hz) in motor cortex. Cortex was modeled as a sheet of neural oscillators that were spatially coupled using a center-surround connection topology. Manipulating the inhibitory surround was found to evoke reliable transitions between synchronous oscillation patterns and traveling waves. These transitions modulated the simulated local field potential in agreement with physiological observations in humans. Intermediate levels of surround inhibition were also found to produce bistable coupling topologies that supported both waves and synchrony. State-dependent perturbation between bistable states produced very rapid transitions but were less reliable. We surmise that motor cortex may thus employ state-dependent computation to achieve very rapid changes between bistable motor states when the demand for speed exceeds the demand for accuracy.

## Introduction

Spatiotemporal waves of electrical activity are a ubiquitous phenomena in the cortex yet the functional relevance of these activity patterns remains unknown (Wu et al., 2008). Task-related waves have been observed in hippocampus (Lubenov and Siapas, 2009), sensory brain regions (Delaney et al., 1994; Arieli et al., 1996; Prechtl et al., 1997), and motor cortex (Rubino et al., 2006). Spontaneous waves have also been observed in cortex in the absence of stimuli (Nauhaus et al., 2009, 2012). Indeed, spatiotemporal wave activity appears so prevalent in the brain that some researchers suggest that it may be a fundamental aspect of neural computation itself (Coombes et al., 2003; Gong and van Leeuwen, 2009).

Waves arise naturally in oscillatory media and nearly all aspects of brain function exhibit some form of oscillatory neural activity (Buzsáaki, 2006; Huang et al., 2010). Neural oscillations can occur at the level of a single neuron's membrane potential or at the network level where it arises from the interplay of recurrently connected populations of excitatory and inhibitory neurons (Wilson and Cowan, 1972). Synchronization of oscillatory activity in the brain has received a great deal of interest as a putative mechanism behind perceptual feature-binding (Eckhorn et al., 1988; Sompolinsky et al., 1990; Singer and Gray, 1995) and long-range neuronal communication (Varela et al., 2001; Fries, 2005). Historically these theoretical accounts have tended to focus on the special case of *in-phase* synchronous locking where the phases of all neural oscillators converge to the same (coherent) value. Yet systems of coupled oscillators, such as weakly coupled neural networks (Hoppensteadt and Izhikevich, 1997), may also synchronize with arbitrary phase shifts (*out-of-phase* locking) depending on the sign and relative strengths of the coupling coefficients (see Pikovsky et al., 2001, for a review). Spatiotemporal wave patterns represent the special case of synchronized out-of-phase locking where the phase shifts between oscillators are proportional to the spatial distance between them. In the present study, we suggest that spatiotemporal waves in motor cortex may play a role in voluntary motor movements.

There is abundant empirical evidence for the role of synchronized oscillations in motor cortex. Voluntary movement production coincides with task-specific increases in the long-range synchronization of beta bandwidth (13–30 Hz) oscillations common to the motor cortex, the pyramidal tract neurons which project from the motor cortex to the spinal motor neurons, as well as the contra-lateral muscles recruited by the movement (Baker et al., 1999). Movement initiation also coincides with a reduction in the observed spectral power of beta bandwidth oscillations in motor cortex (Sanes and Donoghue, 1993; Murthy and Fetz, 1996) followed by a rebound in beta power upon termination of the movement (Neuper and Pfurtscheller, 1996). The cortical idling hypothesis (Pfurtscheller et al., 1996) attributes such transient fluctuations in the spectral power of oscillatory cortical activity to dynamic re-organization of the phases of the underlying neural activity, where low spectral power is thought to reflect functionally active cortical processes and high power is thought to reflect functionally inactive (idle) cortical processes. In these accounts, high and low spectral power regimes are typically equated with synchronized and desynchronized neural activity, respectively (e.g., Pfurtscheller and Lopes da Silva, 1999). In the present study, we consider that equivalent changes in spectral power might also occur when spatially synchronous patterns transition to wave patterns due to the net cancellation of phases in the latter.

Networks of phase-coupled oscillators are ideal for modeling the synchronization of oscillatory neural activity in a simplified mathematical form (Ermentrout, 1994). In this approach, the limit cycle of intrinsically spiking neural activity is reduced to a description of spike timing in terms of the phase of firing alone. The reduction to a phase-only description is valid provided that interactions between coupled oscillators are sufficiently weak not to drive the membrane potential far from the limit cycle so that higher order effects come into play (Hoppensteadt and Izhikevich, 1997). The synchronization characteristics of canonical neural models (type I and type II membrane models) are well described by the phase interaction function which defines how the timing of an oscillator is either advanced or retarded by an external perturbation depending on the time of arrival (Ermentrout and Kopell, 1986; Rinzel and Ermentrout, 1989; Hansel et al., 1995; Ermentrout, 1996; Izhikevich, 1999, 2007). Type I neural models have non-negative phase interaction functions which do not readily lead to synchronization under excitatory (positive) coupling. Conversely, type II neural models have phase interaction functions that span both negative and positive values and always lead to synchronization under excitatory coupling.

The Kuramoto oscillator (Kuramoto, 1984) represents the simplest model of a type II neural oscillator where the phase interaction function is approximated by a sinusoidal function that represents the first mode of a broad class of type II phase interaction functions. Spatially coupled Kuramoto oscillators can be considered a canonical model of oscillatory cortical populations with broad excitatory coupling modulated by a narrow inhibitory surround (Breakspear et al., 2010). Ermentrout and Kleinfeld (2001) showed that Kuramoto oscillators which are spatially coupled using short-range excitatory coupling and long-range inhibitory coupling will produce self-organized patterns of synchrony or waves depending upon the strength of the inhibitory connections. They suggest that synchrony in sensory cortex corresponds to a state of sensory recognition whereas waves represent a state of sensory readiness where the waves serve to periodically modulate the sensitivity of stimulus-specific regions of cortex.

Building on Ermentrout and Kleinfeld (2001), we propose that switching between waves and synchrony in motor cortex may likewise play a role in the onset and offset of motor behavior, although in our proposal the functional assignment of waves and synchrony is reversed. Spatial wave patterns have greater information capacity than synchronous patterns which are highly spatially redundant. This is because the spatially uniform pattern can be reconstructed from the known phase of any oscillator whereas reconstructing a wave pattern requires additional information regarding the direction and wavelength. Even more information is required to reconstruct specific deviations in the pattern from a regular planar wave. We therefore, posit that the morphology of wave patterns may be used to encode distinct movement states in motor cortex and that synchrony encodes for the absence of movement. Voluntary switching between motor movement and motor rest may thus be achieved by switching the motor cortex between patterns of waves and synchrony by manipulating the lateral inhibitory connections in the motor cortex. In physiological terms, we suggest that modulation of the lateral inhibitory connections may be performed by the excitatory glutamatergic neurons that project into the motor cortex from the thalamus and are known to play a role in the initiation of voluntary movement (Alexander and Crutcher, 1990). Glutamate modulates the precise balance of long-range excitatory and inhibitory lateral connections in primary motor cortex. This balance is thought to be crucial to the function of the intrinsic circuitry within the primary motor maps (Keller, 1993; Sanes and Donoghue, 2000).

The present cortical model adopts a center-surround style connection topology rather than sparse long-range inhibitory connections. Center-surround connection topologies are widely regarded as a neurobiologically realistic model of the lateral connectivity in cortex and are known to induce both synchrony and wave patterns in oscillatory neural networks (e.g., Ermentrout, 1998; Ermentrout and Terman, 2010). Center-surround coupling can even support both synchronous and wave solutions simultaneously under identical parameter values (Ermentrout, 1985). By manipulating the strength of the inhibitory surround in the lateral coupling topology, we seek to demonstrate a putative mechanism for controlled switching between distinct cortical states (waves versus synchrony) in an equivalent (small) patch of motor cortex. We also explore the efficacy of anisotropic center-surround coupling topologies for controlling the spatial morphology of the emergent wave patterns. Finally, we provide a framework for relating these findings to observations of oscillating field potentials during repetitive movements in human subjects (Boonstra et al., 2007).

## Results

**Figure 1:**
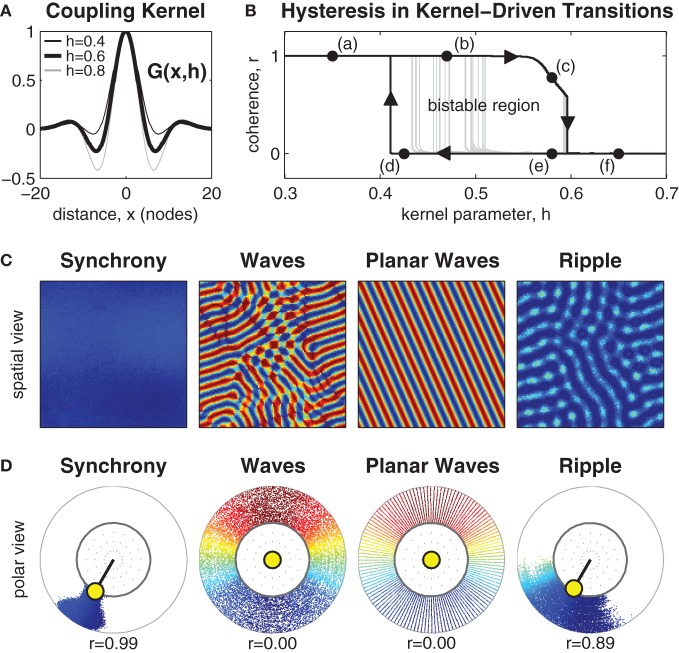
**Manipulating the inhibitory surround evokes spatial synchrony, ripple and wave patterns. (A)** The isotropic coupling kernel in one spatial dimension. The strength of the inhibitory surround is controlled by kernel parameter *h*∈[0,1]. **(B)** Shows hysteresis in the phase coherence (*r*) of the spatial pattern when the strength of the inhibitory surround is slowly manipulated between *h* = 0.3 and *h* = 0.7. Dark line indicates the maximal extent of hysteresis observed. It confirms the existence of bistable patterns for inhibitory surrounds in the range 0.41 ≲ *h* ≲ 0.59. Light gray lines indicate early transitions from waves to synchrony due to heterogeneities in the pattern. **(C)** Exemplar spatial patterns for 128 × 128 oscillators. Color indicates the phase of each oscillator. Synchronous patterns exhibit high phase coherence (*r* ≈ 1) and occupy the upper branch of the hysteresis plot (e.g., labels *a* and *b*). Wave patterns exhibit weak phase coherence (*r* ≈ 0) and occupy the lower branch of the hysteresis plot (labels *d–f*) although only planar waves are stable near the transition boundary (label *d*). Ripple patterns exhibit medium-high coherence (*r* > 0.5) and only occupy the upper branch of the hysteresis plot (e.g., label *c*) where *h* > 0.54. **(D)** The same exemplars shown in polar form using the same color scale. Radial dispersion of the phase points is for visual clarity only. The true radius of every phase point is always *r* = 1 which corresponds to the inner ring. The yellow circle indicates the centroid of all oscillator phases. The polar angle of the centroid corresponds to the mean phase (ψ) and its radial length corresponds to the phase coherence (*r*).

Center-surround coupling was modeled with a smooth coupling kernel *G*(*x, h*) based on the fourth derivative of the Gaussian surface, where *x* is spatial distance and *h*∈[0,1] controls the strength of the inhibitory surround. When *h* = 0 this kernel is exactly Gaussian and represents the spatial distribution of only excitatory lateral connections. When *h* = 1 this kernel equates to the fourth derivative of the Gaussian and represents the recruitment of a ring of inhibitory lateral connections in the local surround while retaining an outer ring of weak excitatory coupling. Manipulating *h*∈[0,1] thus equates to manipulating the recruitment of the inhibitory lateral connections in the local coupling topology (see Figure 1A).

### The effects of isotropic center-surround coupling

Systematic manipulation of parameter *h* revealed that synchronous patterns emerge exclusively for weak inhibitory surrounds (*h* < 0.49) whereas wave patterns emerge exclusively for strong inhibitory surrounds (*h* > 0.59). Exemplar patterns of synchrony and waves are shown in Figure 1 in both the spatial domain and the phase domain. The observed patterns of synchrony and waves are consistent with those of Ermentrout and Kleinfeld (2001) and confirm that center-surround coupling produces equivalent spatial patterns to that produced by short-range excitatory coupling combined with long-range inhibitory coupling.

The nature of the transition boundary between synchrony and waves was explored further by numerical continuation of stable solutions between *h* = 0.3 and *h* = 0.7. The resulting spatial patterns were classified according to phase coherence (*r*) using Kuramoto's (1984) order parameter, where synchronous patterns were identified by high phase coherence (*r* ≈ 1) and wave patterns were identified by low phase coherence (*r* ≈ 0). A definition of the Kuramoto order parameter (Equation 19) is provided in the Methods.

Transitions between synchronous and wave patterns thus correspond to transitions between high and low values of phase coherence. The observed transitions in coherence are shown in Figure 1B where the upper branch represents stable synchronous patterns (marked by labels *a* and *b*) and the lower branch represents stable wave patterns (marked by labels *d–f*). The hysteresis loop in the transitions between synchrony and waves is a hallmark of a *bistable* dynamical system. Here, bistable wave and synchronous solutions are observed for inhibitory surrounds with intermediate strengths (0.41 ≲ *h* ≲ 0.59). Bistable synchronization patterns are of interest because they suggest how a fixed anatomical brain network is capable of supporting distinct, yet stable, brain states.

The extent of the observed bistable region in Figure 1B varies according to the presence of irregularities in the spatial patterns. Wave patterns are especially prone to spatial heterogeneities. Those which are highly regular, such as planar waves, maintain stability nearer to the leftmost transition boundary (e.g., label *d*) than do irregular wave patterns. The latter tend to escape to the synchronous state much earlier, as shown by the light gray trajectories in Figure 1B.

Yet another type of bistable spatial pattern was also observed, which we call *ripple*. Ripple patterns are an intermediate state between waves and synchrony that have the appearance of near-synchronous solutions with small amplitude spatial modulations (see Figure 1). They evolve from synchronous patterns when the strength of the inhibitory surround is increased above *h* ≈ 0.54 and are strongly coherent (0.5< *r* < 1). Ripples occupy the downward sloping branch (c) of the hysteresis loop in Figure 1B. They lose stability above *h* ≈ 0.59 whereupon they collapse to full wave solutions. Although, ripple patterns appear similar to waves in the spatial domain, they are distinct from waves because the phase pattern does not span the entire unit circle. Similar patterns have been previously reported in one-dimensional rings by Kazanci and Ermentrout (2006). Ripple patterns are of interest in the present study because they break the symmetry of the purely synchronous pattern and facilitate transitions away from synchrony toward waves, as will be discussed in the next section.

### Perturbation-driven transitions under bistable center-surround coupling

The existence of bistable wave and synchronous solutions implies that the cortex can support both states using a fixed coupling topology. In this regime, the requirement for a physiological mechanism to modulate the activity of the inhibitory surround would vanish since transitions between waves and synchrony could instead be achieved by direct perturbation of the oscillator phases between the co-existing attractor basins.

### Random perturbation

Random perturbation of the oscillator phases failed to elicit state transitions between waves and synchrony. In light of the growing evidence for state-dependent computation in the brain (Buonomano and Maass, 2009) we instead sought a state-dependent perturbation scheme that could exploit the properties of the current state to push the system toward the alternative attractor basin in a directed manner.

### State-dependent perturbation

We conjectured that a perturbation of each oscillator phase away from the phase of the *mean field* could selectively push the system toward either waves or synchrony in a state-dependent fashion. The mean field (ψ, *r*) is defined by the centroid of all oscillator phases mapped onto the unit circle, where ψ is the mean phase of all oscillators and *r* is the radial length of the centroid (Mardia, 1972). The radial length *r* corresponds to the phase coherence of the oscillator distribution which, as previously mentioned, is markedly distinct for waves (*r* ≈ 0) and synchrony (*r* ≈ 1). The mean field of each exemplar pattern in Figure 1D is indicated by a yellow circle.

A simple state-dependent perturbation scheme was proposed that repels the phase of each oscillator away from the mean field phase by the amount

(1) $$\Delta \theta_{x}=k\sin(\theta_{x}-\psi )$$

where *k* > 0 is the amplitude of the perturbation, θ_*x*_ is the phase of the oscillator at spatial position *x* and ψ is the mean phase of all oscillators. Intuitively, this perturbation scheme breaks the symmetry of the current solution by amplifying deviations from the mean phase to push the system toward the alternative attractor. In mathematical terms, coupling to the mean field is equivalent to all-to-all coupling between oscillators (Kuramoto, 1984; Strogatz, 2000). This perturbation scheme may therefore be realized physiologically by the momentary activation of a dense network of inhibitory inter-neurons that couple all oscillators in the local network. The inhibitory basket cells which extend laterally in motor cortex for more than 1 mm (Keller, 1993) could potentially serve this role. It has previously been shown analytically that such all-to-all inhibitory coupling will destabilize the synchronous state in a one-dimensional ring of oscillators (Kazanci and Ermentrout, 2006). Here, we explore numerically that the same coupling may likewise serve to destabilize waves.

**Figure 2:**
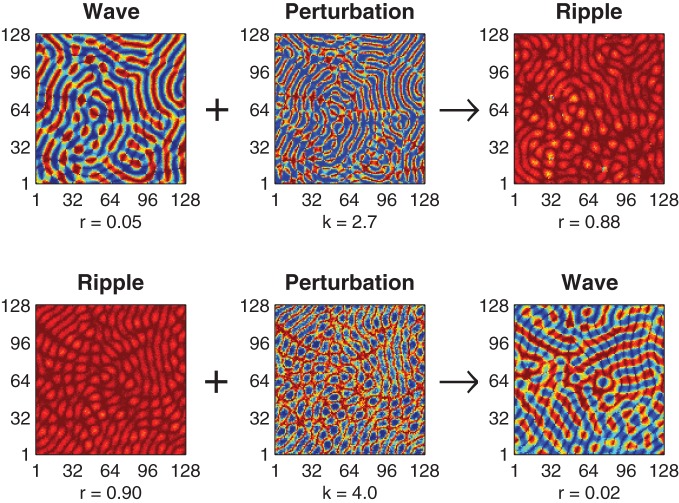
**Perturbation-driven transitions between wave and ripple patterns. Top row** illustrates the application of the state-dependent perturbation (*k* = 2.7) to an initial wave pattern (*r* = 0.05) resulting in convergence to a stable ripple pattern (*r* = 0.88). **Bottom row** illustrates a transition in the reverse direction, namely, perturbation (*k* = 4.0) of a ripple pattern (*r* = 0.90) to a wave pattern (*r* = 0.02). In both cases the connection topology is held fixed (*h* = 0.57) and convergence to the final state is achieved within 500 ms.

The optimal value of the perturbation amplitude parameter *k* depends upon the choice of coupling kernel parameter *h* and the direction of the perturbation. Figure 2 illustrates successful perturbation induced transitions between ripple and wave patterns for a fixed kernel (*h* = 0.57) where the optimal perturbation amplitude for reliable wave-to-ripple transitions is *k* = 2.7 (SE 0.050) and that for ripple-to-wave transitions is *k* = 4.0 (SE 0.051). The optimal perturbation amplitude for each uni-directional transition was determined by first using an adaptive Bayes method (Kontsevich and Tyler, 1999) to estimate the upper and lower limits (*k*_low_ < *k* < *k*_high_) of the perturbation amplitude where state transitions occurred at 50% probability or better—we refer to this range of *k* values as the *transition zone* of the perturbation. The geometric midpoint of the transition zone was then nominated as the optimal perturbation amplitude *k* for the given coupling kernel *h*. The standard error (SE) of the optimal perturbation amplitude was estimated by pooling the SEs of the upper and lower limits of the transition zone (see Methods).

Achieving reliable perturbations from synchrony to waves (not shown) is also possible with this method but these perturbations require very large amplitudes (*k* » 6) in order to amplify the inherently small phase deviations in the synchronous solution enough to break the symmetry of the initial solution.

### Bi-directional state-dependent perturbation

To constrain the choice of parameters in the state-dependent perturbation method, we surveyed the parameter space seeking the optimal combination of *k* and *h* that permitted reliable *bi-directional* transitions between waves and synchrony. This required identifying the coupling kernel configuration for which the optimal perturbation amplitude for the uni-directional transitions was the same in both directions. This constraint immediately excluded coupling kernels that support synchronous patterns since the amplitude of the perturbation required to escape a synchronous solution far exceeds that required to escape a wave solution. The parameter survey was therefore restricted to the range of coupling kernels (0.56 < *h* < 0.59) that support ripple solutions.

**Figure 3:**
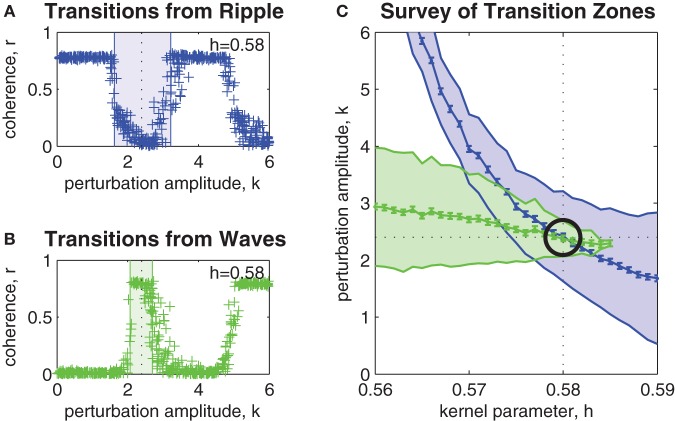
**Parameter survey of the perturbation method. (A)** Shows the effect of manipulating perturbation amplitude (0 < *k* < 6) when perturbing randomly selected ripple patterns within a network coupling topology fixed at *h* = 0.58. Vertical axis represents the phase coherence (*r*) of the resultant pattern at *t* = 4 s post-perturbation. Resultant patterns with *r* > 0.5 were classified as ripple and those with *r* < 0.5 were classified as waves. The shaded blue region indicates the range of perturbation amplitudes (1.6 < *k* < 3.2) where transitions from ripple to waves occurred with 50% success rate or better. We refer to this region as the transition zone. The midpoint of the transition zone (*k* = 2.4) marks the optimal *k* for achieving transitions from ripple to waves for the given network coupling topology. **(B)** Same as above, except here the transitions are from waves to ripple. The transition zone in this case is 2.2 < *k* < 2.6 and the optimal perturbation amplitude is *k* = 2.4. **(C)** Shows the combined transitions zones for both ripple-to-wave (blue) and wave-to-ripple (green) transitions for all coupling kernel configurations in the range 0.56 < *h* < 0.59. The mid-lines of the transition zones indicate the optimal perturbation amplitudes for each kernel *h*. Error bars indicate the standard error of the estimates (all are smaller than ± 0.07). The intersection of the mid-lines (indicated by the black circle) occurs at *h* ≈ 0.58 and *k* ≈ 2.4. It corresponds to the optimal combination of kernel *h* and perturbation *k* parameters for bi-directional perturbations between wave and ripple patterns.

Figure 3A shows the effect of perturbation amplitude on the outcome of perturbation trials applied to randomly selected ripple patterns that arise within a fixed bistable coupling topology (*h* = 0.58). Each cross marks the outcome of a single perturbation trial. As before, resultant spatial patterns with phase coherence *r* < 0.5 were classified as waves. Those patterns with *r* > 0.5 were classified as ripple. In the example shown, the upper and lower limits of the transition zone (shaded blue region) were estimated as *k*_low_ = 1.6 (SE 0.049) and *k*_high_ = 3.2 (SE 0.052). The midpoint of the transition zone *k* = 2.4 (SE 0.051) marks the optimal perturbation amplitude for inducing ripple-to-wave transitions within the given coupling topology.

Similarly, Figure 3B shows the estimated transition zone (shaded green region) for perturbing waves to ripple within the same coupling topology (*h* = 0.58). In this example, the optimal perturbation amplitude for inducing wave-to-ripple transitions is *k* = 2.4 (SE 0.049) which corresponds to the midpoint of *k*_low_ = 2.2 (SE 0.049) and *k*_high_ = 2.6 (SE 0.049).

The survey of all such transition zones across a range of coupling topologies is shown in Figure 3C. It shows the combined transition zones for both ripple-to-wave (blue) and wave-to-ripple (green) transitions for coupling kernel parameters in the range 0.56 < *h* < 0.59. The mid-lines of the transition zones represent the estimates of the optimal perturbation amplitudes *k* for each kernel *h*. The error bars along the mid-line represent the SEs of those estimates and are less than ± 0.07 in all cases. The point where the mid-lines intersect (indicated by the black circle) indicates the optimal choice of parameters (*h* = 0.58, *k* = 2.4) for inducing bi-directional transitions between ripple and waves. This corresponds to evoking bidirectional state transitions between points (c) and (e) in Figure 1B.

### Comparison with human data

The validity of the present model was verified by comparing the time course of state transitions in the simulated local field potential with previously published magnetoencephalogram (MEG) data showing modulated beta oscillations (20–25 Hz) in human motor cortex during a repetitive finger-tapping task (Boonstra et al., 2007). In that experiment, subjects followed ipsilateral auditory cues to isometrically contract their left and right index fingers at a target frequency ratio of 3:5 for multiple trials lasting 45 s at a time. MEG has high temporal resolution but lacks the spatial resolution needed to detect the small spatial wavelengths (approximately 10 mm) reported by Rubino et al. (2006). The comparisons of the model with human data are therefore limited to the time course of the modulations of the simulated field potential during state transitions induced by (1) manipulating the strength of the inhibitory surround in the coupling topology and (2) bi-directional state-dependent perturbation while the coupling topology was held fixed in the optimal bistable regime. Here, the simulated local field potential is a gross approximation of the net membrane potential of synchronized neural activity. We refer to it as the *pseudo field potential* (PFP) to distinguish it as a hypothetical measure (Equation 20 in Methods).

### Amplitude modulation of beta oscillations

**Figure 4:**
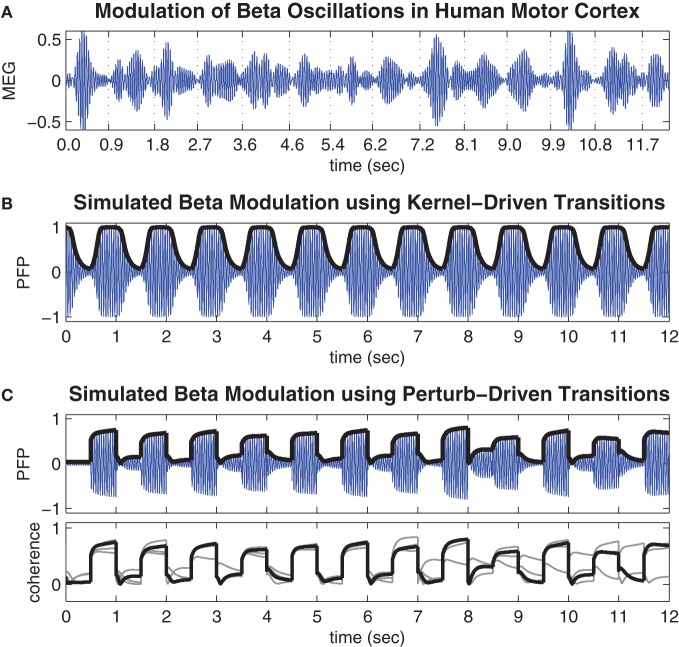
**Movement-related modulations of beta oscillations. (A)** Representative trial of movement-related modulation of beta oscillations in the MEG signal of the contra-lateral hand region of human motor cortex during a repetitive finger tapping task (adapted from Boonstra et al. (2007) and bandpass filtered at 20–25 Hz). Minimal amplitude oscillations tend to coincide with times of peak muscle force which are indicated by the time labels. **(B)** Time course of the pseudo field potential (PFP) generated by the cortical model when spatial coupling was toggled between conditions of weak (*h* = 0.4) and strong (*h* = 0.7) inhibitory surrounds at regular 0.5 s intervals. Heavy line indicates phase coherence (*r*) which corresponds to the amplitude envelope of the PFP by definition. **(C)** Same as above except here the coupling kernel is held fixed in the bistable regime (*h* = 0.58) while the oscillator phases are subjected to state-dependent perturbations (*k* = 2.4) at regular 0.5 s intervals. Lower panel shows the phase coherence for the entire run of 48 s superimposed in 4 × 12 s blocks.

Figure 4A shows a representative time course of beta oscillations (20–25 Hz) in the MEG signal reconstructed from the hand region of human motor cortex by Boonstra et al. (2007). The times of peak muscle force production during the finger-tapping task are indicated by the labels on the time axis. Notice these times tend to coincide with periods of minimal power (amplitude) in the beta oscillation. These observations are consistent with previous reports of task-related beta modulation in motor cortex (Sanes and Donoghue, 1993; Murthy and Fetz, 1996; Pfurtscheller et al., 1996).

Figure 4B shows the time course of the PFP generated by the present model when the spatial coupling kernel was toggled between monostable regimes of synchrony (*h* = 0.4) and waves (*h* = 0.7) at regular 0.5 s intervals. The toggling interval was chosen to be consistent with the finger-tapping task in Boonstra et al. (2007). The resulting PFP exhibits a baseline 22.5 Hz oscillation frequency that reflects the natural frequencies (22.5 ± 0.5 Hz) of the individual oscillators in the model and matches the beta bandwidth (20–25 Hz) oscillations observed in the human motor cortex by design. More importantly, the amplitude envelope of the PFP (shown by the heavy black line in Figure 4B) reflects the phase coherence of the spatial pattern and is an emergent property of the self-organized phase patterns. Specifically, synchronous patterns are highly coherent and therefore yield large amplitude oscillations in the PFP whereas wave patterns are weakly coherent and therefore yield small amplitude oscillations in the PFP. These modulations of the PFP amplitude are qualitatively similar to the task-related modulations of beta oscillations observed in human motor cortex (Figure 4A) yet they do not entail desynchronization as is often supposed to be the case. Furthermore, the observation that low amplitude oscillations tend to occur during motor movement and high amplitude oscillations tend to occur in the absence of movement lends support to our postulate that waves encode motor action states and synchrony encodes motor rest.

Figure 4C shows the time course of the PFP when the coupling topology is held fixed in the bistable regime (*h* = 0.58) while state-dependent perturbations (*h* = 2.4) are applied at regular 0.5 s intervals. These transitions also modulate the amplitude envelope of the PFP in the same qualitative manner as those observed in human motor cortex except here the transitions between states are noticeably faster. This is a known property of bistable systems which, unlike monostable systems, can avoid protracted convergence times by perturbing the state variables directly between co-existing stable attractor basins. However, this speed comes at the expense of reliability since the perturbation-driven transitions do not always succeed, as can be seen in the lower panel of Figure 4C. It shows the amplitude envelope of the PFP for the entire 48 s simulation (superimposed as four 12 s blocks) which contains some examples of perturbations that failed to elicit a successful transition (such as at *t* = 8.5 s). These occasional failures illustrate an inherent variability in the state-dependent perturbation method—that are quite comparable to the empirical data—even though the system itself is entirely deterministic.

We note that the fast time scale of the oscillatory rhythms in the model is naturally defined by the autonomous frequency of the oscillators (22.5 ± 1 Hz). However, the slower time scale, at which synchronization occurs, scales linearly with the magnitude of the coupling weights. Since, the coupling weights were arbitrarily normalized to unity, the absolute transition times between different synchronization states should not be over-interpreted. Nonetheless, the relative transition times of kernel-driven versus perturbation-driven transitions can still be meaningfully compared.

### Trial-averaged spectrograms

To compare the simulations results with physiological data as well as illustrate the difference in average transition times between kernel-driven and perturbation-driven transitions, we followed the methods of Boonstra et al. (2007) to compute the trial-averaged spectrograms of the simulated PFP signals previously shown in Figure 4. These results also confirm that beta power modulation in the present model is consistent with that observed in humans although we stress that the PFP is only a gross approximation of local field potential and should not be over-interpreted.

**Figure 5:**
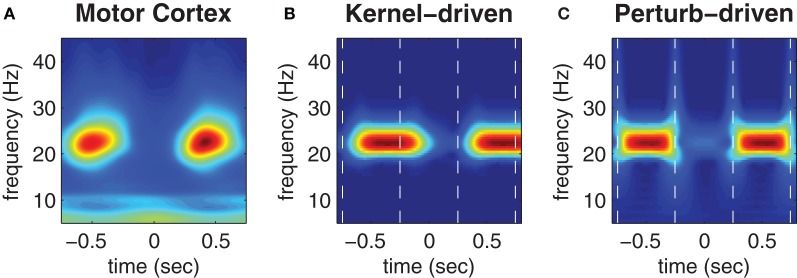
**Trial-averaged spectrograms of state transitions. (A)** Trial-averaged spectrogram of the first principle component (accounting for 50% of the variance) of MEG signals reconstructed from the contra-lateral hand region of human motor cortex during a repetitive finger tapping task (reproduced from Boonstra et al., 2007). **(B)** Trial-averaged spectrogram of the pseudo field potential data presented in Figure 4B. Vertical dashed lines indicate the onsets of kernel transitions. Wave patterns have low spectral power (blue) and synchrony has high spectral power (red). **(C)** Corresponding spectrogram of the pseudo field potential for the perturbation-driven transitions presented in Figure 4C. Dashed lines indicate the perturbation times. Spectral power has a linear color scale in all panels.

Figure 5A (reproduced from Boonstra et al., 2007) shows the trial-averaged wavelet spectrogram of MEG source data reconstructed from the hand region of human motor cortex during the repetitive finger-tapping task. High beta bandwidth power (red) coincides with low muscle force production whereas low beta power (blue) coincides with high muscle force production (muscle force is not shown).

Figures 5B,C show corresponding wavelet spectrograms of the simulated PFP for both the kernel-driven and perturbation-driven state transitions, respectively. The model reproduces the gross modulation of beta power observed in the empirical MEG data in both cases—only the transition times differ. Specifically, kernel-driven transitions (Figure 5B) required approximately 200 ms to converge whereas the perturbation-driven transitions (Figure 5C) appear to be nearly instantaneous. The relative differences in convergence time demonstrates that the method of perturbing the cortical model between bistable states does permit substantially faster transitions than can be achieved by manipulating the coupling topology between monostable regimes.

### The effects of anisotropic coupling

**Figure 6:**
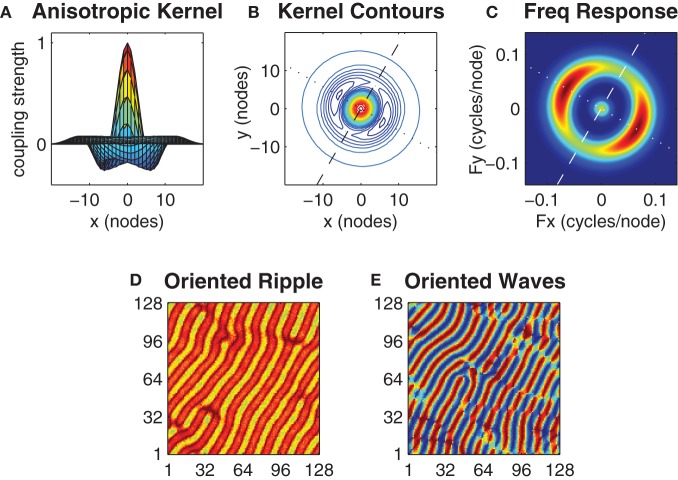
**Anisotropy in the kernel influences the spatial orientation of the wave patterns.** Panels **(A)** and **(B)** show an anisotropic kernel which has weakest inhibitory coupling along the major axis (dashed line in **B**) and strongest inhibitory coupling along the minor axis (dotted line in **B**). The major axis is oriented at β = 60°. Panel **(C)** shows the spatial frequency response of this kernel where color indicates power on a linear scale (blue is minimum, red is maximum). The kernel parameters *h*_0_ = 0.52 and *h*_1_ = 0.64 were chosen so that the peak power response along the minor axis is twice that of the major axis while keeping the mean value of *h*_0_ and *h*_1_ centered on the optimal *h* = 0.58 value previously identified for the case of the isotropic kernel. Panels **(D)** and **(E)** show exemplar ripple and wave patterns that arise with this kernel from near-synchronous and uniform random initial conditions, respectively. Notice the predominant orientation of the wavefronts in both patterns are aligned with the major axis of the kernel.

The isotropic form of the center-surround coupling topology investigated thus far does not constrain the spatial orientation of the emergent wave patterns, which instead show little spatial ordering and change from simulation to simulation. Having assumed that distinct motor actions are encoded by the morphologies of distinct spatial wave patterns we now introduce an anisotropic form of the center-surround coupling topology (illustrated in Figures 6A,B) and show that it evokes wave patterns with a pre-determined spatial orientation. The anisotropic coupling kernel allows the strength of inhibitory surround *h*(α, *h*_0_, *h*_1_) to vary as a function of the angular orientation (α ∈ [0°, 360°]) of the coupling direction where parameter *h*_0_ defines the inhibitory strength along the major axis and parameter *h*_1_ defines the inhibitory strength along the minor (orthogonal) axis of the kernel.

We sought the minimum degree of anisotropy in the kernel that reliably evoked spatially oriented waves without departing far from the optimal bistable coupling (*h* = 0.58) previously identified for the case of the *isotropic* kernel. However, we found no obvious threshold that distinguished the emergence of oriented waves from non-oriented waves since oriented waves were merely slower to emerge when the anisotropy was small. We instead used the kernel's spatial frequency response (Figure 6C) to nominate those kernel parameter values (*h*_0_ = 0.52 and *h*_1_ = 0.64) that produced a 2:1 ratio in the peak spectral power of the minor axis relative to the major axis, while satisfying the constraint that ½(*h*_0_ + *h*_1_) = 0.58 (Figure 6C). Under these constraints, the anisotropic kernel produced both oriented ripple (Figure 6D) and oriented waves (Figure 6E), depending upon initial conditions.

### Stability analysis of planar waves

A linear stability analysis was undertaken to ascertain those conditions under which isotropic coupling supports stable waves and synchrony. The analysis was restricted to the case of planar waves,

(2) θ(x, t)=Ωt+mx,

with wavenumber *m* (cycles/node) and homogeneous oscillator frequencies, Ω (Hz), following the method of Kazanci and Ermentrout (2006) for the one-dimensional ring. Synchrony is represented by the special case of *m* = 0.

The stability of the planar wave was determined by considering the growth in time of the spatial perturbation,

(3) $$\psi (x,t)=e^{inx}e^{\lambda_{n}t},$$

where *n* is the wavenumber of the perturbation and λ_*n*_ is its eigenvalue. The real part of λ_*n*_ is the growth rate of the perturbation and must be non-positive for all *n* for the planar wave to be stable. In the present model, the eigenvalues are readily computed as

(4) $$\lambda_{n}=\text{FT}{\left[J(x,h)\right]}_{n}-\text{FT}{\left[J(x,h)\right]}_{0}$$

where *J*(*x, h*) = *G*(*x, h*) cos(*mx*) is a product of the coupling kernel and *FT* represents the Fourier transform. The homogeneous oscillator frequency, Ω, has no bearing on stability. The derivation of Equation (4) is presented in the Methods.

**Figure 7:**
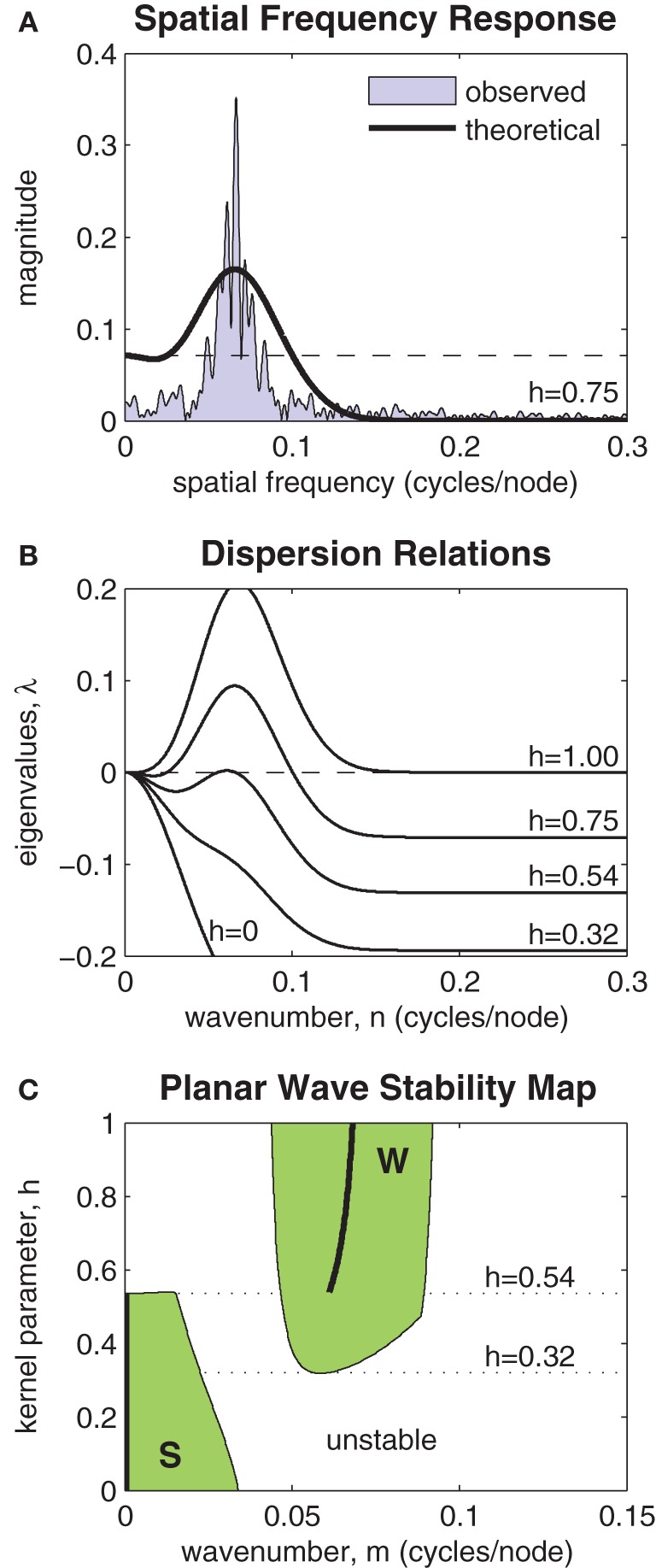
**Stability analysis of planar waves. (A)** Relationship between the Fourier transform of the coupling kernel (heavy line) and the observed spatial frequencies (shaded) of a typical solution of the model. The horizontal dashed line indicates the magnitude of the zero'th Fourier component of the kernel. The largest Fourier component above this line predicts the frequency of the emergent spatial pattern. **(B)** Dispersion relations show the stability of a given planar wave solution (*m* = 0.064) to perturbations at various spatial frequencies *n* ∈ [0,0.3]. Perturbations with any λ > 0 are unstable. Dispersion relations for selected kernel parameter values are shown superimposed. In this example, the planar wave is stable for all kernels with *h* < 0.54. **(C)** Stability map showing which planar waves *m* ∈ [0,0.15] are stable for kernel parameters *h* ∈ [0,1]. The shaded region marked “S” represents stable synchronous and near-synchronous solutions (*m* ≈ 0). The region marked “W” represents stable wave solutions. Kernels with 0.32 < *h* < 0.54 support both waves and synchrony in a bistable fashion. The heavy black line predicts the frequency of the stable wave pattern that is most likely to emerge from synchronous initial conditions.

Figure 7A highlights the relationship between the Fourier transform (FT) of *J*(*x, h*) and the spatial frequency of the patterns observed in the model. A typical spectrum of spatial frequencies in a one-dimensional ring of oscillators is shown by the shaded region marked “observed.” The Fourier coefficients of *J*(*x, h*) are shown by the heavy black curve labeled “theoretical.” The horizontal dashed line indicates the magnitude of the zero'th Fourier coefficient. The largest Fourier coefficient above that line represents the most unstable spatial mode of the perturbation. It is that mode which grows fastest and predicts the dominant spatial wavelength of the emergent wave pattern. If all Fourier coefficients of *J*(*x, h*) are smaller (more negative) than the zero'th coefficient then the planar wave is stable against all perturbations.

The spatial stability is formalized by the so-called *dispersion relations* shown in Figure 7B. These plot the eigenvalues of a range of spatial perturbations (*n* ∈ [0,0.3]) applied to a planar wave with a given wavenumber. In this example, the wavenumber was specified as *m* = 0.064. The dispersion relations for a range of selected kernels (*h* = 0, 0.32, 0.54, 0.75, 1) are shown. The planar wave is only stable for those dispersion curves that are non-positive for all *n*. In this example, that corresponds to coupling kernels with *h* < 0.54. The emergence of instabilities with non-zero wavenumber (*m* = 0.065) indicates that stability is lost via a sub-critical Turing bifurcation.

Figure 7C extends the analysis by mapping the stability of all planar waves with wavenumbers *m* ∈ [0, 0.15] for all kernel parameters *h* ∈ [0,1]. Two distinct regions of stability are observed (shaded green). The region marked S represents stable synchronous and near-synchronous solutions that have small spatial frequencies (*m* < 0.041). The region marked W represents stable planar waves with spatial frequencies in the range 0.044 < *m* < 0.091. There is a clear region of overlap between these regions where coupling kernels with 0.32 < *h* < 0.54 support both waves and synchrony in a bistable fashion. Within the bistable zone, near-synchronous patterns are restricted to spatial wavelengths of 43.5 nodes or greater (*m* < 0.023) whereas wave patterns are restricted to wavelengths of between 11.1 and 21.7 nodes (0.046 < *m* < 0.090). The heavy black line in Figure 7C tracks the spatial frequency of the most unstable component of the purely synchronous solution (*m* = 0). It predicts the frequency of the stable wave pattern that is most likely to emerge from synchronous initial conditions for a given kernel *h* ∈ [0,1].

## Discussion

Spatiotemporal synchronization patterns in cortex were modeled using an array of Kuramoto phase oscillators that were spatially coupled using a neurobiologically inspired center-surround coupling topology. Controlled switching between self-organized patterns of waves and synchrony was achieved by manipulating the strength of the inhibitory connections in the coupling topology. This controlled switching reproduced the characteristic fluctuations in the spectral power observed in the beta bandwidth (20–25 Hz) oscillations in human motor cortex during a finger tapping task (Boonstra et al., 2007). Specifically, the simulated local field potential of the model exhibited high beta power during synchronous solutions and attenuated beta power during spatiotemporal wave solutions. The cortical idling hypothesis (Pfurtscheller et al., 1996) attributes such fluctuations in spectral power to switching between synchronized versus desynchronized modes of oscillatory activity in cortex. Yet in this case the simulated cortical activity was never actually desynchronized but was synchronized either as spatially coherent patterns or as wave patterns. These results emphasize that spatial phase locking of neural oscillators can involve much richer synchronization patterns than is usually considered under the traditional notions of global synchrony versus desynchrony.

### The functional role of waves and synchrony

We propose that spatiotemporal wave solutions represent active states in motor cortex whereas synchrony represents inactive motor states. This proposal finds support on information theoretic grounds because spatial wave patterns afford greater information capacity than do spatially synchronous patterns. The general agreement between the simulation results and experimental observations of attenuated beta power during movement production (Sanes and Donoghue, 1993; Murthy and Fetz, 1996) followed by a rebound of beta power upon movement termination (Neuper and Pfurtscheller, 1996; Boonstra et al., 2007) gives some empirical validity to these assumptions. The pathology of Parkinson's disease provides further support for these assumptions where pathological synchrony of beta oscillations in the basal ganglia thalamocortical motor loop is known to prevent the initiation of voluntary movements (Schnitzler and Gross, 2005). Disrupting that pathological synchrony restores voluntary movement (Tass, 2003).

We suggest that switching spatially synchronous oscillatory neural activity to a specific spatiotemporal wave pattern may be governed by excitatory thalamocortical projections modulating the lateral inhibitory connections within the motor cortex. Voluntary movements are known to be initiated in cortex when these thalamocortical projections are dis-inhibited by the basal ganglia which is itself implicated in movement selection (see Alexander and Crutcher, 1990). Our simulation results with anisotropic coupling topologies also suggest that the spatial morphology of the emergent wave patterns may likewise be governed by modulating the lateral inhibitory connections in the motor cortex. The local coupling topology in motor cortex can thus be manipulated to control not only switching between waves and synchrony but also to control the spatial wavelength and orientation of the wave patterns that emerge. The spatial morphology of such patterns could plausibly be discriminated by the pyramidal tract neurons which descend from the motor cortex and innervate the motor neurons within the spinal cord. The dendritic tree of pyramidal neurons has recently been shown capable of actively discriminating spatiotemporal patterns of synaptic input, including the speed and direction of propagating waves of activity (Branco et al., 2010). In this way, distinct spatiotemporal patterns in motor cortex may thus be translated into distinct patterns of muscle movements.

### The role of bistability

Bistable cortical processes have previously been implicated in spontaneous switching between two distinct modes of alpha activity (Freyer et al., 2011). We considered that bistability might also play a role in motor control and we explored the feasibility of motor cortex using a bistable coupling topology as a means of evoking very rapid onset and offset of movements. Bistable dynamical systems can potentially achieve rapid transitions between co-existing stable states through perturbation of the state variables although the nature of the perturbation is crucial when many state variables are involved, as is the case here. We proposed a state-dependent perturbation scheme that repels each oscillator from the global mean field of the current solution state. This perturbation scheme serves to push wave solutions toward synchrony and vice versa, thus permitting bidirectional transitions between waves and synchrony with the same form of perturbation. The perturbation is mathematically equivalent to an instantaneous burst of inhibitory coupling between all neural oscillators in the cortex.

The optimal bistable coupling topology (*h* = 0.58) for bidirectional perturbation (*k* = 2.4) supports co-existing wave and ripple solutions rather than waves and synchrony. The ripple patterns represent a branch of near-synchronous solutions with small spatial variations that break the symmetry of the synchronous pattern and facilitate the effects of the state-dependent perturbation. Like synchronous patterns, ripple patterns exhibit strong spectral power in the simulated local field potential which we equate with the state of motor inaction. Switching between waves and ripple modulates the simulated local field potential in a comparable manner to that observed when switching between waves and synchrony.

Simulation confirmed that repeated perturbation trials are capable of producing sequential transitions between wave and ripple patterns and that these transitions converge significantly faster than those achieved by toggling the coupling topology between monostable dynamical regimes. However, transition speed comes at the cost of reliability since not all perturbation trials successfully induce a state transition.

The general shape of the coupling kernel in the bistable regime seems biologically plausible. For isotropic coupling, bistability is observed in numerical simulation for *h* ∈ [0.41, 0.59] and analytically for *h* ∈ [0.32, 0.54]. The two findings are quite consistent even though the analytical estimate is based on a one-dimensional homogeneous simplification of the numerical model. In particular, the one-dimensional analysis is likely to exaggerate the extent of the lower bound of bistability since it ignores the differing responses of isotropic coupling along the two spatial axes of the planar wave. We also note that the discrepancies in the upper bound are due to the exclusion of ripple patterns from the stability analysis since ripples are not wave solutions. In this regard, the numerical simulations show that ripples are onset at *h* ≈ 0.54 which is in perfect agreement with the analytic prediction for the loss of waves.

### Conclusions

Ermentrout and Kleinfeld (2001) originally proposed distinct functional roles for waves and synchrony in sensory brain regions where synchrony was equated with a state of perceptual recognition and waves were equated with a state of perceptual scanning. We extend that general proposal and offer an account of the functional roles of waves and synchrony in motor control where we posit that waves in motor cortex encode motor actions and synchrony encodes the absence of motor action. These functional assignments are the reverse of those proposed by Ermentrout and Kleinfeld (2001) since here we equate synchrony with inactive cortical processes as suggested by the cortical idling hypothesis (Pfurtscheller et al., 1996). Specifically, we posit that the distinct spatial morphologies of wave patterns in motor cortex can encode distinct motor action states which, we suggest, may be decoded (discriminated) by the pyramidal tract neurons which descend from the motor cortex and innervate the motor neurons in the spinal cord.

Numerical simulation supports our functional assignments of waves encoding motor action and synchrony encoding motor rest. Controlled switching between self-organized patterns of waves and synchrony in the cortical model reproduces the general character of task-dependent fluctuations of beta bandwidth oscillations observed in the local field potential of human motor cortex. Such task-dependent fluctuations are widely interpreted as dynamic reorganizations of the phases of the neural oscillatory activity underlying the local field potential. This reorganization is typically envisaged as a shift between in-phase synchronization and desynchronized modes of operation. This view is exemplified by the cortical idling hypothesis, however, it lacks a theoretical account of the neural mechanism behind these modes of cortical synchronization. Nor does it explain how the cortex might encode information within the desynchronized state. The present model demonstrates that task-dependent fluctuations of the local field potential can be achieved by switching cortex between modes of spatial synchrony and spatiotemporal wave patterns without resort to desynchronization.

The present model also offers a theoretical neural mechanism by which information may be encoded within the morphology of the spatial synchronization patterns. Numerical simulation demonstrates that the spatial morphology of the wave patterns can be manipulated by using an anisotropic local coupling topology to potentially encode a variety of motor movement states within the same patch of motor cortex. We suggest the laterally spreading inter-neurons of the motor cortex may likewise be modulated by cortico-thalamic projections that selectively enable a target motor action as instructed by the basal ganglia. The motor cortex may also exploit bistable cortical topologies to evoke rapid transitions between motor rest and the target motor action. In this case, fast-onset instructed-delay movements may be primed in motor cortex as a bistable ripple pattern (representing the motor ready state) which is later perturbed into a full wave pattern causing the target movement to unfold rapidly. However, such rapid transition speed comes at the cost of reliability since perturbation does not always induce a successful transition from ripple to wave. Perturbation between bistable cortical states may therefore only be a feasible mechanism for achieving rapid changes in brain states when the demand for speed exceeds the demand for accuracy. Whether the motor control system actually adopts such a strategy is an open question.

Spatially-coupled phase oscillator models are appropriate models to study the spatiotemporal synchronization patterns of oscillatory cortical activity such as that observed in motor cortex during movement production and motor preparation. In such cases, the spatial extent of the lateral coupling topology determines the spatial scale of the emergent patterns thus experimental observation of such patterns in cortex depends crucially on the spatial resolution of the recording sites. We anticipate that cortical oscillatory activity which appears to be desynchronized at course spatial resolution will likely reveal fine spatiotemporal synchronization structure when observed at higher spatial resolutions. Recent findings that the dendritic tree of cortical pyramidal neurons can actively discriminate the speed and direction of sequences of synaptic input suggests that the spatial resolution of wave-like synchronization patterns may even occur at scales below that of the network (Branco et al., 2010). In future studies we plan to investigate further our assertion that the morphology of spatial wave patterns may be decoded by the pyramidal cells that descend from the motor cortex by modeling the pyramidal cells as coincidence detectors that can act as matched filters of specific spatiotemporal activity patterns.

## Methods

Motor cortex was modeled as a two-dimensional sheet of non-locally coupled Kuramoto (1984) oscillators

(5) $$\frac{\partial \theta (x,t)}{\partial t}=\omega (x)-\int_{{\mathbb{R}}^{2}}G(|x-x\prime |)\sin(\theta (x,t)-\theta (x\prime ,t))dx\prime$$

where θ(*x, t*) is the instantaneous phase of the oscillator at spatial position *x* ∈ ℝ^2^, ω(*x*) is its natural frequency and *G*(|*x* − *x*′|) denotes the spatial coupling kernel. Two forms of kernel were investigated—an *isotropic* form and an *anisotropic* form. The isotropic kernel was defined as

(6) $$G(z)=e^{-bz^{2}}+4he^{-bz^{2}}\left(\frac{1}{3}b^{2}z^{4}-bz^{2}\right)$$

where *z* = |*x* − *x*′| represents spatial distance and parameter *h* ∈ [0,1] dictates the strength of the kernel's inhibitory surround. When *h* = 0 the kernel is purely Gaussian (with slope *b*) and thus has no inhibitory (negative) coupling. When *h* = 1 the kernel corresponds to the fourth derivative of the Gaussian which has a strong inhibitory surround and an outer ring of weak excitatory coupling. For intermediate values of *h* the kernel is a mixture of the Gaussian and the fourth derivative of Gaussian (see Figure 1A).

The *anisotropic* form of the kernel was also defined by Equation (6) except in this case the fixed *h* parameter was redefined as a 2 π-periodic function of radial position relative to the kernel origin,

(7) $$h(\alpha )=\frac{1}{2}(h_{0}-h_{1})\cos(2(\alpha -\beta ))+\frac{1}{2}(h_{0}+h_{1}),$$

where α is the orientation angle of (*x* − *x*′) in polar coordinates and β is the orientation angle of the major axis of the anisotropic kernel. Parameters *h*_0_, *h*_1_ ∈ [0,1] define the strength of the inhibitory surround along the major and minor (orthogonal) axes of the kernel, respectively (see Figures 6A,B).

### Stability analysis of planar waves

The linear stability of planar wave solutions,

(8) θ(x, t)=Ωt+mx,

of the isotropically coupled system with homogeneous oscillator frequencies, ω(*x, t*) = Ω, was analyzed following the method of Kazanci and Ermentrout (2006). The spatial orientation of the planar waves was ignored because of rotational invariance. The spatial frequency of the wave along the axis of propagation was described in one dimension by the wavenumber *m* (cycles/node). The corresponding spatial wavelength being 2 π/*m* (nodes). Equations (5) and (6) were therefore treated as a one-dimensional ring of oscillators for the purpose of stability analysis.

Stability of the wave solution was determined by considering the growth in time of some spatial perturbation ψ(*x, t*) applied to the wave,

(9) θ(x, t)=Ωt+mx+ψ(x, t).

Substituting Equation (9) into (5) yields

(10) $$\begin{array}{c} \frac{\partial \psi (x,t)}{\partial t}=-\int G(|x-x\prime |)H(mx-mx\prime +\psi (x,t)\\ -\psi (x\prime ,t))dx\prime \end{array}$$

where *H*(θ) = sin(θ) is the Kuramoto phase interaction function. We note that the same analysis generalizes to any odd 2π-periodic phase interaction function that satisfies *H*(−θ) = −H(θ). Substituting variables, *y* = *x* − *x*′, allows Equation (10) to be rewritten as

(11) $$\frac{\partial \psi_{x}}{\partial t}=-\int G(y)H(my+\psi_{y}-\psi_{x-y})dy,$$

where the *t* subscripts have been omitted for brevity. Taylor series expansion,

(12) $$H(my+\psi_{y}-\psi_{x-y})=H(my)+H\prime (my)[\psi_{y}-\psi_{x-y}]+O({\left[\psi_{y}-\psi_{x-y}\right]}^{2})$$

allows the small non-linear terms in [ψ_*y*_ − ψ_*x*−*y*_] to be ignored. Substituting Equation (12) back into (11) yields the linearized growth rate of the perturbation as

(13) $$\frac{\partial \psi_{x}}{\partial t}=-\int G(y)H(my)dy-\int G(y)H\prime (my)[\psi_{y}-\psi_{x-y}]dy,$$

where ∫ *G*(*y*) *H*(*my*) *dy* = 0 because *G*(*y*) is even and *H*(*y*) is odd. For the case of the Kuramoto phase interaction, *H*′(*my*) = cos(*my*).

Since, Equation (13) is a linear differential equation in ψ, we apply the well-known solution,

(14) $$\psi_{x}=e^{inx}e^{\lambda_{n}t},$$

where *n* is the spatial wavenumber of the perturbation and λ_*n*_ is its eigenvalue. The real part of the eigenvalue represents the growth rate of the perturbation. Substituting Equation (14) in (13) gives

(15) $$\frac{\partial }{\partial t}[e^{inx}e^{\lambda_{n}t}]=-\int G(y)H\prime (my)[e^{inx}e^{\lambda_{n}t}-e^{in(x-y)}e^{\lambda_{n}t}]dy$$

which simplifies to

(16) $$\lambda_{n}=\int G(y)H\prime (my)e^{-iny}dy-\int G(y)H\prime (my)e^{-i0y}dy=\text{FT}{\left[J(y)\right]}_{n}-\text{FT}{\left[J(y)\right]}_{0}$$

where *J*(*y*) = *G*(*y*) *H*′(*my*) and *FT* represents the Fourier transform.

Equation (16) thus describes the growth rate of a spatial perturbation (with wavenumber *n*) applied to a planar wave state (with wavenumber *m*). The planar wave is stable provided Real(λ_*n*_) < 0 for all *n*. Loss of stability results in a Turing bifurcation of the spatial pattern in which case the most unstable wavenumber of the perturbation tends to dominate the new pattern that emerges (see Cross and Greenside, 2009). This is readily computed for all *n* simultaneously using the FT for a given *m*. The special case of *m* = 0 represents the spatially uniform synchronous state. Dispersion curves for a given planar wave *m* were obtained by plotting λ_*n*_ versus *n*. The stability map of all planar wave solutions was obtained by determining the stability of each wavenumber *m* separately for a range of kernel parameters *h* ∈ [0,1].

### Numerical integration

For computational efficiency, Equation (5) was reformulated as

(17) $$\frac{\partial \theta }{\partial t}=\omega +\cos(\theta )\int_{{\mathbb{R}}^{2}}G(|x-x\prime |)\sin(\theta )dx\prime -\sin(\theta )\int_{{\mathbb{R}}^{2}}G(|x-x\prime |)\cos(\theta )dx\prime$$

by applying the trigonometric identity sin(*u* − *v*) = sin *u* cos *v* − cos *u* sin *v* and then integrated in the form

(18) $$\frac{d\vec{\theta }}{dt}=\vec{\omega }+\cos(\vec{\theta })[\vec{G}*\sin(\vec{\theta })]-\sin(\vec{\theta })[\vec{G}*\cos(\vec{\theta })]$$

using a variable time step Runge–Kutta method where $\vec{\theta }$ and $\vec{\omega }$ are both (*m* × *n*) matrices, $\vec{G}$ is a (*p* × *q*) matrix of kernel coupling coefficients and ^*^ denotes two-dimensional convolution. In all cases, the size of the oscillator array was fixed at (*m* × *n*) = (128 × 128) with wrapped boundary conditions. The size of the kernel was fixed at (*p* × *q*) = (41 × 41) with a Gaussian full-width-half-height of eleven nodes (i.e., *b* = −4 log(0.5)/11^2^). The natural oscillator frequencies $\vec{\omega }$ were randomly selected from a Gaussian distribution with a mean of 22.5 Hz and standard deviation of 0.5 Hz.

### Numerical continuation

Hysteresis curves (Figure 1) were computed by numerical continuation of steady-state solutions by stepping the kernel *h* parameter from *h* = 0.4 to *h* = 0.7 (and vice versa) by a fixed increment (Δ *h* = 0.001) upon convergence of the current step. The convergence criterion was satisfied when the root-mean-square of the instantaneous $d\vec{\theta }/dt$ values fell below 0.2 rad/s.

### Phase metrics

The instantaneous phase coherence *r*(*t*) and mean phase ψ(*t*) of the oscillator array were computed by equating the real and imaginary parts of Kuramoto's (1984) complex order parameter,

(19) $$r(t)e^{i\psi (t)}=\int_{{\mathbb{R}}^{2}}e^{i\theta (x,t)}dx,$$

at a given time *t*. Geometrically, the values *r* and ψ correspond to the radius and phase of the centroid of the oscillator phases when mapped onto the unit circle.

### Pseudo field potential

The PFP was defined as the sum of the cosine components of the oscillator phases. It serves as a gross approximation of the local field potential for an equivalent patch of cortex by regarding the cosine of oscillator phase as analogous to membrane voltage potential. It can also be expressed exclusively in terms of *r*(*t*) and ψ(*t*) by substituting Euler's formula into Equation (19) and equating the real parts to obtain

(20) $$\text{PFP}(t)=\int_{{\mathbb{R}}^{2}}\cos(\theta (x,t))dx=r(t)\cos(\psi (t)).$$

### Spectrograms

Spectrograms of the time-varying PFP signal (Figure 4) were computed by the same method as Boonstra et al. (2007) using a continuous wavelet transform with a complex Morlet wavelet (*F*_*c*_ = 5, *F*_*b*_ = 1) that was scaled from 5 to 45 Hz using regular 0.5 Hz increments. Sampling frequency was 1000 Hz.

### State-dependent perturbation

The state-dependent perturbation applied to each oscillator phase was defined as

(21) Δθ(x,t)=ksin(θ(x,t)−ψ(t))

where *k* > 0 is the amplitude of the perturbation and ψ(*t*) is the mean phase of all oscillators. This perturbation repels each oscillator away from the centroid of the phase field. For the case of ripple patterns, the radial position of the centroid is shifted from *r* > 0.5 toward *r* ≈ 0. For the case of full wave patterns, the radial positions shifts from *r* ≈ 0 toward *r* > 0.5 in the anti-phase orientation. This form of perturbation may therefore be used to induce bi-directional transitions between ripple and waves for an appropriate choice of perturbation amplitude parameter *k*.

### Bayesian estimation of the transition zones

We separately estimated the optimal values of perturbation amplitude *k* for achieving uni-directional transitions from both (1) ripple to waves and (2) waves to ripple for a range of kernel parameters 0.56 ≤ *h* ≤ 0.59. The phase coherence *r* of the oscillator array was measured at *t* = 4 s post-perturbation to quantify the outcome of each perturbation trial. Outcomes with phase coherence *r* > 0.5 were classified as ripple and those with *r* < 0.5 were classified as waves. These outcomes (waves versus ripple) toggle periodically as a function of *k* due to the circular nature of phase perturbations (see Figures 3A,B). We estimated the lower and upper bounds of the smallest values of *k* need to achieve a state transition at 50% probability. We called this range of *k* values the *transition zone* of the perturbation.

The upper and lower transition boundaries were estimated separately by fitting a transition probability curve with the logistic form

(22) Prob(transition|k)=1/(1+exp(−β×(k−α)))

where α is the 50% threshold of the logistic curve and β is its slope. The slope was fixed at β = 10 following pilot studies. Fixing β reduced the variability in the final α estimates. The α estimates were obtained by adaptive Bayesian estimation using the Psi method (Kontsevich and Tyler, 1999) as implemented by the Palamedes Psychometric Toolbox for Matlab (Prins and Kingdom, 2009). A total of *n* = 20 perturbation trials were used to fit each logistic curve. Efficiency of each Psi run was improved by specifying normally distributed α priors (with unity standard deviation) that were centered on the α estimate previously obtained for the neighboring kernel *h* value. Initial conditions were randomized on each trial and transients were given *t* = 4 s to converge prior to the onset of the perturbation.

We nominated the mid-line of the transition zone as the optimal *k* value to achieve uni-directional transitions for a given kernel *h*. The point at which the mid-line of the ripple-to-wave transition zone crossed the mid-line of the wave-to-ripple transition zone marked the optimal kernel configuration *h* = *h*_opt_ for achieving *bi-directional* transitions using the same perturbation amplitude *k* = *k*_opt_. The SEs of the mid-lines were estimated by pooling the SEs of the upper and lower bounds of the transition zones, respectively (see Figure 3C).

### Tuning the anisotropic kernel

We sought the minimal level of asymmetry needed in the anisotropic coupling kernel (Figures 6A,B) to achieve spatial wave patterns with a predetermined orientation. Starting with the optimal bistable symmetric kernel (*h* = *h*_opt_) we manipulated the degree of discrepancy Δ *h* between the anisotropic kernel parameters by setting *h*_0_ = *h*_opt_ − Δ *h* and *h*_1_ = *h*_opt_ + Δ *h* for a range of Δ *h* ∈ {0.00, 0.01, 0.02,…} values. The spatial frequency response spectrum of the kernel (Figure 6C) was computed using the two-dimensional discrete FT with 1024 × 1024 samples. The frequency response of the kernel predicts the spatial frequency and orientation of the spatial wave patterns. However, the frequency responses change smoothly with Δ *h* so there is no obvious choice for the minimal anisotropy. We therefore arbitrarily chose that value of Δ *h* which produced a spatial frequency response spectrum whose peak power along the kernel minor axis was twice that of the major axis at the matching radial frequency band.

### Conflict of interest statement

The authors declare that the research was conducted in the absence of any commercial or financial relationships that could be construed as a potential conflict of interest.
